# TLR7 Activation Accelerates Cardiovascular Pathology in a Mouse Model of Lupus

**DOI:** 10.3389/fimmu.2022.914468

**Published:** 2022-07-04

**Authors:** Ahmed S. Elshikha, Xiang Yu Teng, Nathalie Kanda, Wei Li, Seung-Chul Choi, Georges Abboud, Morgan Terrell, Kristianna Fredenburg, Laurence Morel

**Affiliations:** ^1^ Department of Pathology, Immunology, and Laboratory Medicine, University of Florida, Gainesville, FL, United States; ^2^ Department of Pharmaceutics, Zagazig University, Zagazig, Egypt

**Keywords:** lupus, cardiovascular disease, TLR7, mouse models, autoimmunity

## Abstract

We report a novel model of lupus-associated cardiovascular pathology accelerated by the TLR7 agonist R848 in lupus-prone B6.*Sle1.Sle2.Sle3* (TC) mice. R848-treated TC mice but not non-autoimmune C57BL/6 (B6) controls developed microvascular inflammation and myocytolysis with intracellular vacuolization. This histopathology was similar to antibody-mediated rejection after heart transplant, although it did not involve complement. The TC or B6 recipients of serum or splenocytes from R848-treated TC mice developed a reactive cardiomyocyte hypertrophy, which also presents spontaneously in old TC mice as well as in TC.*Rag^-/-^* mice that lack B and T cells. Each of these cardiovascular lesions correspond to abnormalities that have been reported in lupus patients. Lymphoid and non-lymphoid immune cells as well as soluble factors contribute to lupus-associated cardiovascular lesions in TC mice, which can now be dissected using this model with and without R848 treatment.

## Introduction

Cardiovascular disease (CVD) is one of the leading causes of death in systemic lupus erythematosus (SLE) (1, 2), especially five years post diagnosis (3). SLE patients suffer from a greater risk for CVD, which presents with diverse manifestations including pericarditis, myocarditis, valvular disease, atherosclerosis, thrombosis and arrhythmias. This clinical heterogeneity likely reflects a complex etiology as well as the contribution of multiple risk factors. Widespread use of imaging tools has revealed a high frequency of microvascular impairment and myocarditis in SLE patients (4–8), the majority of which does not lead to clinical presentation (9–11). Prevailing evidence suggests the presence of a coronary microvascular dysfunction in SLE patients, which has emerged as a mechanism of myocardial ischemia, heart failure and arrhythmias distinct from obstructive atherosclerosis. This subtle pathology may contribute to the high incidence of heart failure in SLE patients (12).

Mechanistic and target-identification studies have been hampered by the paucity of murine models of SLE-associated CVD (13). Atherosclerosis induced by a high fat diet in Apoe-deficient mice was worsened in the presence of lupus-susceptibility genes such as the *gld* mutation (14), as well as loci derived from the NZM2410 lupus-prone strain (15, 16). Spontaneous lupus models have been rarely used due to a low penetrance of cardiac lesions (17), except for (NZW x BXSB.*Yaa*)F1 male mice that develop anti-phospholipid syndrome, as well as myocardial lesions and infarction (18). The translocation of X-linked *Tlr7* and *Tlr8* to the Y chromosome in the Y-autoimmune accelerator (*Yaa*) locus leads to a severe lupus-like disease in *Yaa*-associated strains (19, 20). Tlr7-deficiency protected Apoe-deficient mice form high-fat diet induced atherosclerosis (21). Moreover, topical applications of resiquimod (R848), a TLR7/8 agonist that induces a lupus-like disease in non-autoimmune mice (22), triggered the rapid development of myocarditis and dilated cardiomyopathy in non-autoimmune CFN mice but in none of their parental strains, B6, FVB and NOD (23). This phenotype was transferable by splenocytes and serum from R848-treated CFN mice.

These studies suggested that TLR7 signaling may accelerate CVD on specific genetic backgrounds. TLR7 plays a critical role in the pathogenesis of lupus through the production of type I IFN (24). Genetic studies have associated *TLR7* variants with SLE (25) and multiple murine models support a requirement for TLR7 in the development of autoantibodies and subsequent autoimmune pathology (26, 27). We tested the hypothesis that acute TLR7 activation by R848 may induce cardiovascular pathology in lupus-prone mice. We selected the B6.*Sle1.Sle2.Sle3* triple congenic (TC) strain that expresses three lupus susceptibility loci that are necessary and sufficient to induce disease on a B6 non-autoimmune background. TC mice spontaneously develop lupus manifestations such as anti-dsDNA IgG and immune-complex mediated glomerulonephritis (28). The *Yaa* locus (29) or *Tlr7* transgene (30) accelerated disease in monocongenic B6.*Sle1* mice, indicating a synergy between *Tlr7* overexpression and *Sle1*. Cardiovascular phenotypes have however not been evaluated in these mice. Here, we showed that TLR7 activation induced myocytolysis in both lupus-prone and control mice, but a microvascular inflammation of the heart was observed only in lupus-prone TC mice, with features similar to antibody-mediated rejection (AMR). We characterized the cardiovascular lesions and the systemic immune response in these mice with and without TLR7 activation. To dissect the mechanisms responsible for these phenotypes, we used adoptive transfers of serum and splenocytes, as well as TLR7 activation in lymphocyte-deficient mice and myeloid cell-depleted mice. With the results obtained, we propose a model combining acute TLR7 treatment in young TC mice and spontaneous development in aged TC mice to better understand the mechanisms of subclinical microvascular impairment and reactive cardiac hypertrophy in SLE patients and improve their disease prevention or management.

## Materials and Methods

### Mice and Procedures

TC and TC.*Rag2^-/-^* (TC.*Rag^-/-^*) mice have been described previously (28, 31). B6 and B6.*Rag2^-/-^* (B6.*Rag^-/-^*) mice were originally purchased from the Jackson Laboratory (Bar Harbor, ME). All mice were bred and maintained at the University of Florida in specific pathogen-free conditions. Both males and females were used with gender and age-matched controls for each experiment. TC mice either between 9 and 16 weeks of age, before they produce anti-dsDNA IgG (referred to as “pre-autoimmune”), or at 10 months of age, when they have developed autoimmune pathology (referred to as “autoimmune”), were treated with 100 μg resiquimod (R848; Tocris, Minneapolis, MN) in 100 μl acetone (Thermo Fisher Scientific, Waltham, MA) by topical application to the right ear three times a week for 2 weeks. Age-matched B6 mice were treated at the same time. Both males and females were used with gender-matched controls for each experiment. In one experiment, pre-autoimmune TC mice were intravenously injected with 100 µg of clodronate or PBS-loaded control liposomes (Clodrosome, FormuMax Scientific, Sunnyvale, CA) five times over three weeks before the start of the R848 treatment. Tissues were harvested one week later or at the indicated time. For adoptive transfer experiments, splenocytes and serum obtained from TC mice one week after the 2-week R848 treatment were transferred into B6 and TC recipient mice according to a published protocol (23). Briefly, splenocytes were stimulated for 48 h with 5 mg/ml concanavalin A (Sigma-Aldrich, Saint Louis, MO), washed thoroughly with phosphate buffered saline (PBS, Thermo Fisher Scientific) and 2 x 10^7^ cells were injected intravenously into healthy recipients. Pooled serum was injected intra-peritoneally at 200 μl/week for 3 weeks. Recipient mice were sacrificed 6 weeks after the first injection. To measure the heart weight to tibia length ratios, hearts were excised and washed in ice cold PBS, followed by 5% KCl (Sigma-Aldrich) in PBS, then weighed. Tibias were placed in 1N NaOH (Thermo Fisher Scientific) overnight at 37°C, then tibial length was measured with a caliper (World Precision Instruments, Sarasota, FL). This study was carried out in accordance with the guidelines from the Guide for the Care and Use of Laboratory Animals of the Animal Welfare Act and the National Institutes of Health. All animal protocols were approved by the Institutional Animal Care and Use Committee of the University of Florida (IACUC 202009466).

### Autoantibody and Cytokine Measurements

Serum autoantibodies were detected by ELISA. Detection of anti-dsDNA IgG was performed with sera diluted 1:100 as previously described (32). The same ELISA protocol was used with plates coated with 1 μg/ml β2GPI/APOH (R&D systems, Minneapolis, MN), 75 µg/mL cardiolipin (Sigma-Aldrich) in ethanol, 30 µg/ml RNA isolated from mouse splenocytes, or 1 µg/ml mouse heart lysate (Novus, Centennial, CO) to detect IgG with the corresponding specificities in sera diluted 1:50, as previously described (33).

### Heart and Kidney Histology

Hearts were excised after *in situ* perfusion with 10 ml ice-cold PBS through the apex of the left ventricle and processed for histology with hematoxylin and eosin (H&E) as well as trichrome stains. The following histopathologic changes were evaluated and scored on a 1 to 4 scale: vascular congestion, interstitial capillary injury, vascular dilatation, myocytolysis, myocyte vacuolization as minimal (1), mild (2), moderate (3), or severe (4) using papillary muscle involvement to drive scoring. Histopathologic changes were scored as minimal (1) if there was less than 30% of the heart muscles (left and right ventricles and interventricular septum) were involved without involvement of papillary muscles; mild (2) with greater than 30% of the heart muscles involved without involvement of the papillary muscles; moderate-mild (3) with focal involvement of papillary muscles; and severe (4) with a diffuse involvement of the heart muscle and papillary muscles. Myocardial hypertrophy was scored on a scale of 1-3. Mild myocardial hypertrophy was considered with minimal reactive cellular hypertrophy of the left ventricle (1); moderate myocardial hypertrophy was considered as reactive cellular hypertrophy of the left and right ventricle and interventricular septum (2); and severe myocardial hypertrophy corresponded to reactive cellular hypertrophy involving all heart muscles including papillary muscles with obvious luminal narrowing (3). Intravascular inflammation was considered focal if there was scattered inflammation within interstitial vessels without involvement of the papillary muscles (score of 1) and diffuse if inflammation involved the heart muscles including the papillary muscle (score of 2). All scoring was performed by a pathologist (KF) in a blinded manner.

Paraffin-embedded heart sections were also stained by immunohistochemistry for CD45 (1:25 dilution; 30F11) and CD43 (1:25, S7) both from BD Biosciences (Franklin Lakes, NJ). Immunofluorescence analyses of frozen heart sections were performed using antibodies against CD3 (145-2C11, 1:25), B220 (RA3-6B2, 1:25), CD11b (M1/70, 1:25), F4/80 (BM8, 1:25), all purchased from BD Biosciences, CD43 (S7, 1:25), C4d (1:50, Hycult Biotech, Wayne, PA), IgG2a (Southern Biotech, Birmingham, AL, 1:50) and C3 (MP Biomedicals, Irvine, CA; 1:50). Images were captured with a CCD camera connected to an EVOS microscope (Thermo Fisher) and quantitated with ImageJ (nih.gov).

### Flow Cytometry

Single-cell suspensions were prepared using standard procedures from spleens. Single-cell suspensions were prepared from heart tissue by digestion in a mixture of 2mg/mL Collagenase IV (Worthington Biochemical) and 1.2 units/mL Dispase II (Sigma-Aldrich) in perfusion buffer for 45 minutes at 37° C with trituration every 15 minutes, filtered through a 70 µm filter and diluted in 15 ml perfusion buffer, centrifuged at 200 G for 20 min, washed with 1x HBSS (Sigma-Aldrich), centrifuged again and resuspended in 250 µL of 2% FBS/HBSS before FACS staining.

Cells were stained in FACS staining buffer (2.5% FBS, 0.05% sodium azide in PBS). Fluorochrome-conjugated antibodies are as follows: B220 (RA3-6B2), BCL-6 (K112-91), CD11b (M1/70), CD11c (HL3), CD3e (145-2C11), CD62L (MEL-14), CD95 (Jo2), IgD (217-170), Ly6C (gb11), CD40 (3/23), CD80 (16-10A1), CD19 (eBio1D3), CD8a (53-6.7), Ly6G (1A8), IgMb (AF6-78), CD21 (7E9), CD23 (B3B4), CD93 (AA4.1), and T-BET (eBio4B10) were purchased from BD Biosciences. CD4 (RM4-5), CD138 (281-2), MHCII (M5/114.15.2) and IFN-γ (XMG1.2) were purchased from BioLegend (San Diego, CA). CD4 (GK1.5), CD44 (IM7), CD25 (PC61.5), CD69 (H1.2F3), Foxp3 (FJK-16S), GL-7 (GL-7), PD-1 (RMP1-30), and PDCA-1 (eBio927) were purchased from eBioscience (San Diego, CA). Dead cells were excluded with fixable viability dye (eFluor780 or LIVE/DEAD™ Fixable Yellow Dead Cell Stain Kit; Thermo Fisher Scientific). Intracellular staining was performed with a fixation/permeabilization kit (eBioscience). For cytokine detection, splenocytes were stimulated with Leukocyte Activation Cocktail (BD Biosciences) at 37 ^0^C for 4 h. All samples were acquired on an LSRFortessa flow cytometer (BD Biosciences) and analyzed with FlowJo software (Tree Star, Woodburn, OR). Gating schemes for B and T cells are shown in **Figure S1**, and for myeloid cells in **Figure S2**.

### Gene Expression

One week after treatment, 20 - 30 mg of the middle part of perfused hearts was homogenized in RLT buffer (Qiagen, Germantown, MD) with 2% beta mercaptoethanol (Thermo Fisher Scientific) then digested with proteinase K (Roche, Indianapolis, IN) for 10 min at 55 °C and centrifuged. Total RNA was extracted with the RNeasy Mini Kit (Qiagen) and used for qRT-PCR using the High-Capacity cDNA Reverse Transcription Kit (Thermo Fisher Scientific). SYBR Green Dye (BioRAD, Hercules, CA) was used for quantification on the Bio-Rad CFX connect system. The primers used are shown in **Table S1**. Gene expression was quantified with the 2^–ΔΔ^ **^Ct^** method relative to *Ppia* (cyclophilin A).

### Statistics

Statistical analyses were performed with the Graphpad Prism 9.0 software. Differences between groups were evaluated by one-way/two-way ANOVA with correction for multiple tests, or unpaired or paired t tests, as indicated in the text. Unless specified, all tests are two-tailed. Results were expressed as means ± standard deviation. The levels of statistical significance were set at *: *P* < 0.05, **: *P* < 0.01, ***: *P* < 0.001 and ****: *P* < 0.0001.

## Results

### TLR7 Activation Induced an Acute Microvascular Inflammation in the Heart of Lupus-Prone TC Mice

The R848 treatment that has been described to induced autoimmune manifestations in non-autoimmune mice (22) was applied to pre-autoimmune female TC mice and B6 controls (**Figure S3A**). A rapid weight loss and 50% mortality (**Figures S3B, C**) were observed in TC but not in B6 mice, with massive skin hemorrhages (**Figure S3D**) by the end of the third week in. A heavy morbidity has been reported in the related lupus-prone NZM2328 mice after two weeks with the same R848 protocol (**Figure S3D**) (34), but their cardiovascular phenotypes were not evaluated. To be able to characterize the cardiovascular lesions in TC mice before they present these terminal manifestations, we used the protocol previously used in CFN mice (23), with three R848 applications per week for two weeks, and phenotypes evaluated at week 3 (**Figure S3E**). Both male and female TC mice tolerated this regimen with no weight loss or mortality (**Figure S3F**).

**Figure 1:**
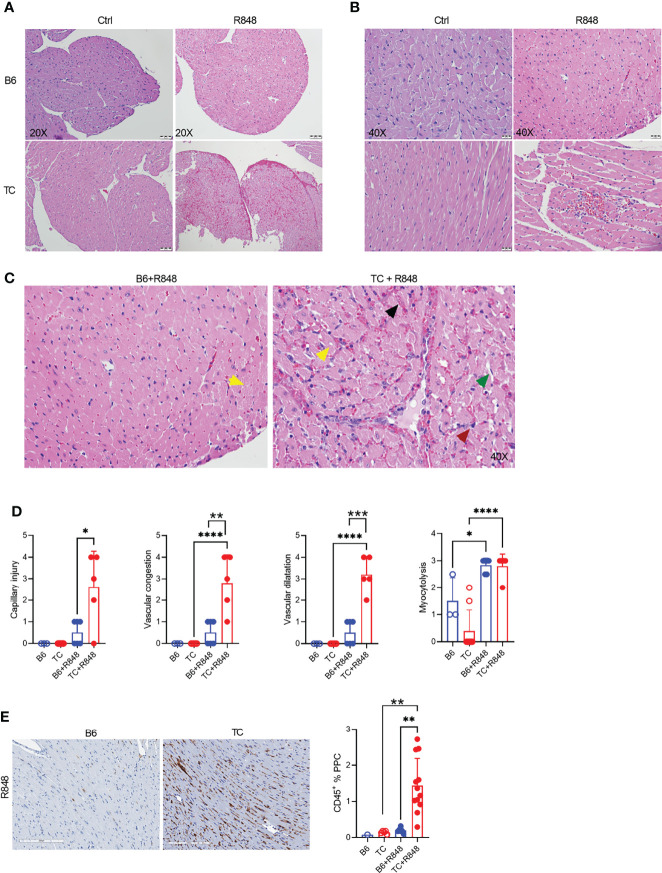
TLR7 activation induces cardiovascular pathology in TC mice. Representative H&E-stained heart sections of untreated and R848-treated B6 and TC mice at 20X **(A)** and 40X **(B)** magnification (scale bars: 50 um and 20 um, respectively) at week 3. **(C)** Representative H&E staining from R848-treated B6 and TC mouse at 40X showing in the TC mouse: vascular congestion (black arrow); vascular dilatation (green arrow); myocytolysis with vacuolization (yellow arrow); and capillary injury with enlarged endothelial cell (red arrow). Capillary injury **(D)**, vascular congestion, vascular dilatation and myocytolysis scores respectively, R848-treated B6 mice were used as a control. **(E)** CD45 staining with representative sections on the left (20X magnification, scale bar: 200 um) and quantitation of the right (PPC: pixel per cell). n = 1 - 5 untreated mice, n = 5 - 12 treated mice per strain. t tests and 1-way ANOVA with multiple comparison tests, * P < 0.05, ** P < 0.01, *** P < 0.001; **** P < 0.001.

The hearts of R848-treated TC but not B6 mice showed on low magnification myocardial tissue with extensive vascular dilatation and congestion as well as red blood cell extravasation (**Figure 1A**). A higher magnification showed enlarged, hyperchromatic endothelial cells indicative of interstitial capillary injury (**Figures 1B, C**). Inflammatory cells were largely confined to the distended lumens of capillaries and venules, with little infiltration into myocytes. Semi-quantitative scoring showed that the congestion with intra-capillary activated leukocytes and interstitial capillary injury was more severe in treated TC than in B6 mice (**Figure 1D**). The heart of treated mice from both strains also presented a mild to moderate myocytolysis and vascular dilatation (**Figure 1D**), which corresponds to degenerative cardiomyocyte damage visible as lightly stained areas in an otherwise homogenously stained myocardium, along with intracellular vacuolization, devoid of lymphocyte infiltration (**Figure 1C**). The heart from untreated mice from either strain showed no microvascular injury and myocytolysis (**Figures 1A, B**). Finally, there was little if any fibrosis in either strain (data not shown).

**Figure 2:**
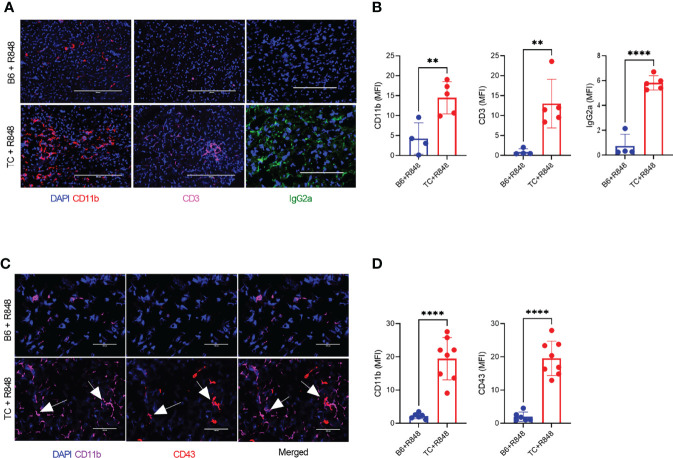
TLR7 activation induces immune cell infiltration in the heart of TC mice. **(A)** Representative heart sections stained for CD11b, CD3 and IgG2a (20X scale bars: 200 um). **(B)** Quantification of CD11b^+^ and CD3^+^ cells as well as IgG2a deposits (MFI, mean florescence intensity). **(C)** Representative heart sections co-stained with CD11b and CD43 (40X, scale bars: 50 um). **(D)** Quantification of CD11b^+^ and CD43^+^ infiltrates (MFI). Arrows indicate co-staining. n = 5 - 12 treated mice per strain. t tests, ** P < 0.01, **** P < 0.001.

The leukocyte infiltration in the heart of treated mice was further characterized by immunohistochemistry and immunofluorescence. Treated TC mice showed extensive homogenous CD45^+^ hematopoietic cell infiltrates (**Figure 1E**), which were more abundant in females than in males (**Supplementary Figure 3G**). Very few, if any, CD45^+^ cells were found in the hearts of untreated pre-autoimmune B6 and TC mice (**Supplementary Figure 3H**). The infiltrates were largely composed of CD11b^+^ with CD3^+^ T cell foci (**Figures 2A, B**). CD19^+^ B cells were not detected (data not shown). The CD11b^+^ and CD3^+^ infiltrates were much less abundant in the hearts from treated B6 mice. Collectively, the presence of microvascular injury and CD11b+ in the heart of R848-treated TC mice are similar to AMR injury that can be seen in heart transplantation (35). We therefore assessed the presence of IgG2a, which forms pathogenic immune complexes that are promoted by TLR7 activation (36). Abundant IgG2a deposits were present in the heart of treated TC but not B6 mice (**Figures 2A, B**). C4d and C3 depositions are surrogate markers of complement activation, especially C4d for AMR (37) and C3 for lupus nephritis, including in the TC model for the latter (38). There was however no detectable complement C4d and C3 deposition in the heart, although both were readily detectable in the kidneys of the same mice (data not shown). This result suggests that complement-independent mechanisms might be involved in the heart of R848-treated TC mice. CD43 expression is the hallmark of CD11b^+^ patrolling monocytes (PMo) that drives TLR7-induced lupus nephritis (39). CD43^+^ cells were present in the heart of treated TC mice (**Figures 2C, D**), and many of them were CD11b-positive (**Figures 1C, D**), suggesting that they correspond to PMos. CD43^+^CD11b^+^ cells were found in TC mice after one week of treatment, suggesting it is an early event in pathogenesis (**Supplementary Figure S3J**). CD43^+^CD11b^+^ cells were also present in the heart of treated B6 mice although in much lower numbers, but not in the heart of untreated young TC mice (**Supplementary Figures S3I-K**). CD43^+^ CD11b-negative cells may reflect the presence of activated T cells in the heart of R848-treated TC mice (**Figures 2C, D**), since CD43 is also a marker of T cell activation (40). Overall, these results show that acute TLR7 activation induced microvascular injury with abundant myeloid infiltrates and antibody deposit that was more severe in TC mice than in B6 controls. This suggested that the TC autoimmune genetic background synergized with TLR7 activation to induce cardiovascular injury.

### Adoptive Transfers of Splenocytes and Serum From R848-Treated TC Mice Induced Myocytolysis Leading to Reactive Myocardial Hypertrophy

**Figure 3:**
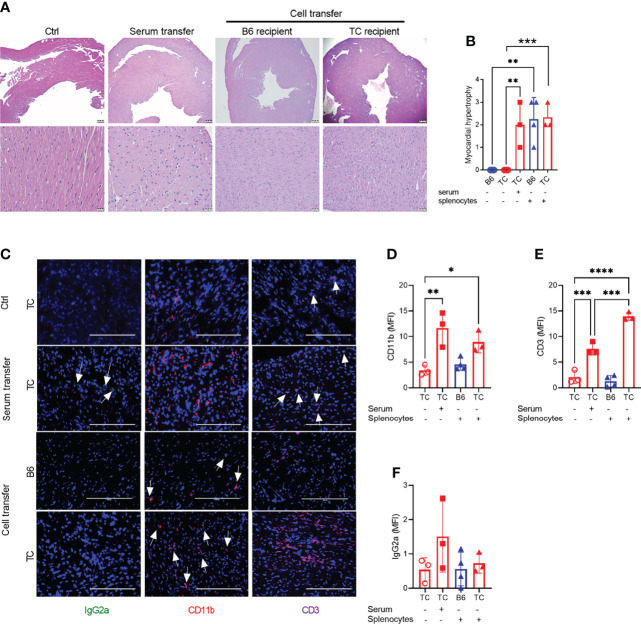
Splenocytes or serum from R848-treated TC mice induced a reactive cardio-hypertrophy in B6 and TC recipients. **(A)** Representative H&E-stained heart sections of the indicated groups (top: 4X, scale bars 200um, and bottom: 40X, scale bars 20 um). **(B)** Myocardial hypertrophy scores. **(C)** Representative heart sections from TC controls as well as TC recipients of serum or splenocytes, and B6 recipients of splenocytes stained for IgG2a, CD11b and CD3 (20X, scale bars: 200 um). Quantification (MFI) of CD11b^+^ **(D)** and CD3^+^ **(E)** cells and IgG2a deposits **(F)**. n = 3 - 4 per group. 1-way ANOVA with multiple comparison tests, * P < 0.05, ** P < 0.01, *** P < 0.001; **** P < 0.001.

**Figure 4:**
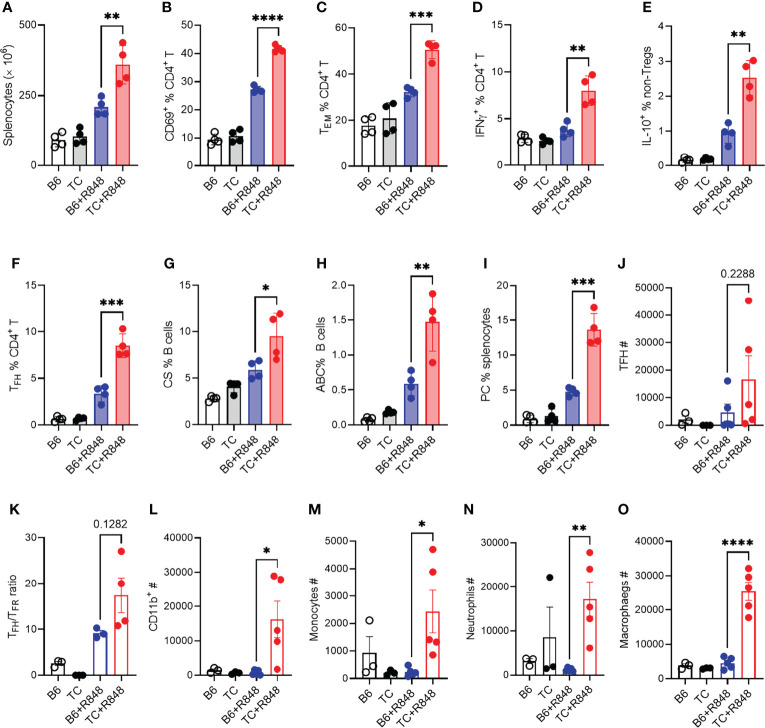
TLR7 activation enhanced lymphocyte activation in TC mice. B6 and TC mice were treated with R848 for 1 week and their splenic CD4^+^ T cell and B cell phenotypes were compared to untreated age-matched control mice. **(A)** Splenocyte numbers. Frequency of CD4^+^ T cells expressing CD69 **(B)**, presenting the CD62L^-^CD44^+^ Tem phenotype **(C)**, expressing IFNγ **(D)** or IL-10 excluding Foxp3^+^ cells, **(E)**. Frequency of PD1^hi^CXCR5^+^BCL-6^+^ Tfh cells **(F)**, CD19^+^IgM^-^IgD^-^class-switched (CS) B cells **(G)**, TBET^+^CD11c^+^ B cells ABCs, **(H)** CD138^+^B220^lo^ plasma cells PC, **(I)**. In another experiment, B6 and TC mice were treated with R848 for 2 week and sacrificed at the end of week 3. T cells and myeloid cells phenotypes in the heart were compared to untreated control mice. **(J)** Number of Tfh cells; **(K)** Ratio of Tfh to Tfr cells; Numbers of CD11b^+^ cells **(L)**, monocytes **(M)**, neutrophils **(N)**, and macrophages **(O)**. n = 3 - 5 per group. *t* tests, *: P < 0.05, **: P < 0.01, ***: P < 0.001. ****: P < 0.0001.

**Figure 5:**
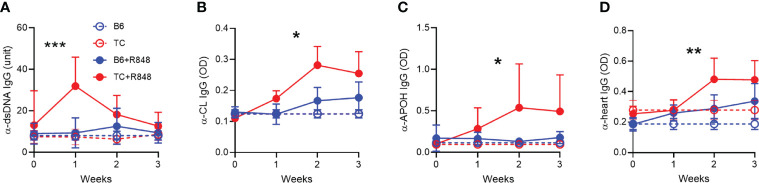
TLR7 activation induced the production of anti-cardiovascular autoantibodies in TC mice. Serum IgG in control (n = 3) and R848-treated B6 and TC mice (n = 11 - 16) directed against dsDNA **(A)**, CL **(B)**, APOH **(C)** and heart tissue **(D)**. Statistics compare treated TC and B6 mice with *t* test between week 1 values **(A)**, and 2-way ANOVA for all values up to week 3 **(B–D)** *: P < 0.05, **: P < 0.01, ***: P < 0.001.

Since R848 directly affects endothelial cells (41), we next investigated whether R848 affected heart tissues directly or indirectly through cellular and/or humoral immune system activation. Splenocytes and serum collected at week 3 from treated TC mice were transferred to untreated B6 (cells only) or TC mice. Untreated and treated mice constituted negative and positive controls, respectively. Six weeks after transfer, both splenocytes and serum transfer from R848-treated TC mice induced a moderate to severe myocardial hypertrophy in both B6 and TC mice. Interstitial capillary injury was not observed (**Figures 3A, B**). Transfer of R848 itself from the donor to the recipient was unlikely since tissues were harvested from donors one week after the last treatment. CD11b^+^ myeloid and CD3^+^ T cell infiltrates, as well as a weak IgG2a deposition were detected in serum recipients (**Figures 3C-F**). Stronger CD3^+^ infiltrates with weak CD11b^+^ myeloid infiltrates but no IgG2a deposition were found in the TC but not the B6 recipients of recipients splenocytes (**Figures 2C** to **3F**). Since the pre-autoimmune untreated recipient TC mice present minimal immune cell activation and autoantibody production (**Figures 4**, **5**), these results showed that reactive myocardial hypertrophy can be induced by either activated immune cells or serum, which contains, in addition to autoantibodies, cytokines and chemokines that may also be involved in tissue injury. There was no difference in cardiovascular phenotypes between the B6 and TC recipients of TC-treated splenocytes (**Figure 3B**), although the cellular infiltrates were lower in B6 recipients. This suggests that overactivated immune cells after R848 treatment, play a greater role in induction of reactive cardiac hypertrophy than the recipient heart’s response.

### TLR7 Activation Induced a Stronger Immune Activation in TC Spleens and Hearts

The induction of cardiac hypertrophy by the serum or splenocytes from R848-treated TC mice suggested that the cardiovascular pathology in R848-treated TC mice may result at least in part from their enhanced immune response to TLR7 activation. We therefore compared the immune response to R848 treatment in the spleen of TC and B6 mice. Splenocyte activation peaked after the first week of treatment and plateaued for the next two weeks (data not shown). We therefore compared the TC and B6 responses in the splenic phenotypes that were altered by 1-week treatment (see **Supplementary Figure S1 and S2** for gating strategy). R848-treatment resulted in increased splenocyte numbers in TC as compared to B6 mice (**Figure 4A**). In addition, a higher frequency of CD4^+^ T cells expressed the CD69 activation marker (**Figure 4B**), differentiated into the effector memory phenotype (Tem) (**Figure 4C**), and produced IFNγ and IL-10 (**Figures 4D, E**) in TC mice.

Next, we investigated the immune infiltrates by flow cytometry response in the heart at week 3 as well as in untreated controls. The number of follicular helper T (Tfh) cells was increased by the R848 treatment, without difference between the two strains (**Figure 4J**). However, the ratio of TFH to follicular regulatory T (Tfr) cells was higher in TC treated mice compared to treated B6 (**Figure 4K**), suggesting a less controlled helped to antibody production by B cells. There was also a profoundly enhanced myeloid response in the hearts of R848 treated TC mice which comprised total CD11b^+^ myeloid cells (**Figure 4L**) and key myeloid subsets, including monocytes, neutrophils, and macrophages (**Figures 4M–O**). No difference was observed between strains in untreated mice, in which the number of myeloid cells was very low. These data confirmed the histology findings (**Figure 2**), showing a greater inflammatory cell infiltrate into the hearts of R848 treated TC mice compared to B6 treated mice.

The heart of treated TC mice showed a higher expression of *Ifng* and *Tnfa* (**Figures S4A, B**), which suggests an infiltration of inflammatory T cells, in agreement with the presence of CD3^+^ infiltrates (**Figures 2A, B**) and the expansion of activated T cells in the spleen. The follicular helper T (Tfh) cell subset was also expanded (**Figure 4F**). Accordingly, treated TC mice presented a higher frequency of class-switched B cells (**Figure 4G**), although the frequency of germinal center B cells was similar between the two strains (data not shown). This is potentially due to TLR7 activation favoring extra-follicular class-switching (42). The frequency of TBET^+^ CD11c^+^ age-related B cells (ABCs), which have been associated with autoantibody production, and plasma cells was also greatly expanded by R848 treatment in TC mice (**Figures 4H, I**). Overall, these results showed that R848 induced a greater response in the adaptive immune system of TC mice.

We then evaluated the effect of R848 on autoantibodies. One week after exposure to R848, TC mice produced a burst of anti‐dsDNA IgG that was not sustained (**Figure 5A**). The production of anti-RNA IgG was modestly increased at week 1 to a similar level in both strains (data not shown). Antibodies directed against cardiolipin (CL) and APOH caused vascular damage in the anti-phospholipid syndrome. Anti-CL and anti-APOH IgG levels increased up to week 2 and were sustained by week 3 in treated TC mice (**Figures 5B, C**). In addition, treated TC mice produced IgG directed against heart tissue (**Figure 5D**). Treated B6 mice produced these antibodies at levels similar to or slightly higher than baseline in untreated mice.

**Figure 6:**
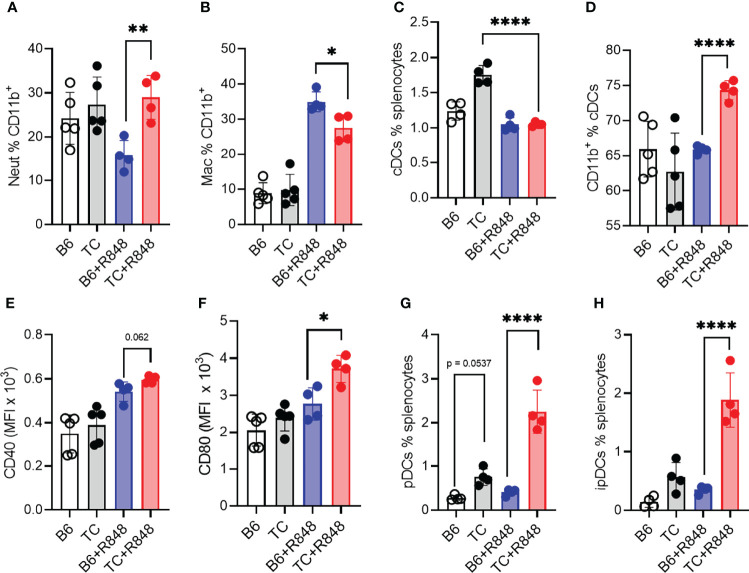
TLR7 activation enhanced myeloid cell activation in TC mice. B6 and TC mice were treated with R848 for 1 week and their splenic myeloid cell phenotypes were compared to untreated age-matched control mice. Frequency of neutrophils **(A)** and macrophages **(B)** in CD11b^+^ cells. Frequency of cDCs in splenocytes **(C)** and CD11b^+^ cells in cDCs **(D)**. Expression of CD40 **(E)** and CD80 **(F)** on cDCs. Frequency of pDCs **(G)** and ipDCs **(H)**. n = 4 - 5 per group. 1-way ANOVA, with multiple comparison tests, *: P < 0.05, **: P < 0.01, ****: P < 0.0001.

Since myeloid cells dominated the infiltrate in the heart of R848-treated TC mice (**Figures 2A, B**), we further characterized the myeloid splenic populations. The frequency of CD11b^+^ splenocytes was similar between treated and untreated mice from both strains but, as expected, from their increased number of splenocytes (**Figure 4A**), treated TC mice had a higher number of CD11b^+^ splenocytes (data not shown). Among them, the frequency of CD11b^+^Ly‐6G^+^ neutrophils was higher in TC mice but not expanded by R848 (**Figure 6A**). In contrast, the frequency of CD11b^+^F4/80^+^ macrophages was expanded by R848, but to a lower extent in TC mice (**Figure 6B**) and the frequency of MHC-II^+^CD11c^+^ conventional DCs (cDCs) was decreased by the treatment in both strains (**Figure 6C**). However, cDCs in treated TC mice shifted to a more inflammatory CD11b^+^ cDC2 phenotype (**Figure 6D**), and showed higher expression of co-stimulation markers CD40 and CD80 (**Figures 6E, F**). Plasmacytoid DCs (pDCs) play a critical role in lupus by producing type I IFNs (43). Untreated TC mice presented an expanded population of CD11b^−^CD11c^+^PDCA‐1^+^ pDCs that greatly increased with R848 treatment (**Figure 6G**). Activated pDCs lose B220 expression to become ipDCs that secrete high amounts of type I IFN (44). The frequency of ipDCs was much higher in the spleen of treated TC mice (**Figure 6H**). A corresponding higher production of type I IFN was confirmed by a higher expression of IFN-inducible genes (ISGs) in the heart (**Supplementary Figures S4C, D**). Together, these results showed that R848 expanded and activated both myeloid and lymphoid cells. This was further enhanced by the lupus-prone genetic background, which supported the production of pathogenic autoantibodies in TC mice that is consistent with the observed vascular and cardiac damage.

### Spontaneous Heart Lesions in Autoimmune TC Mice

**Figure 7:**
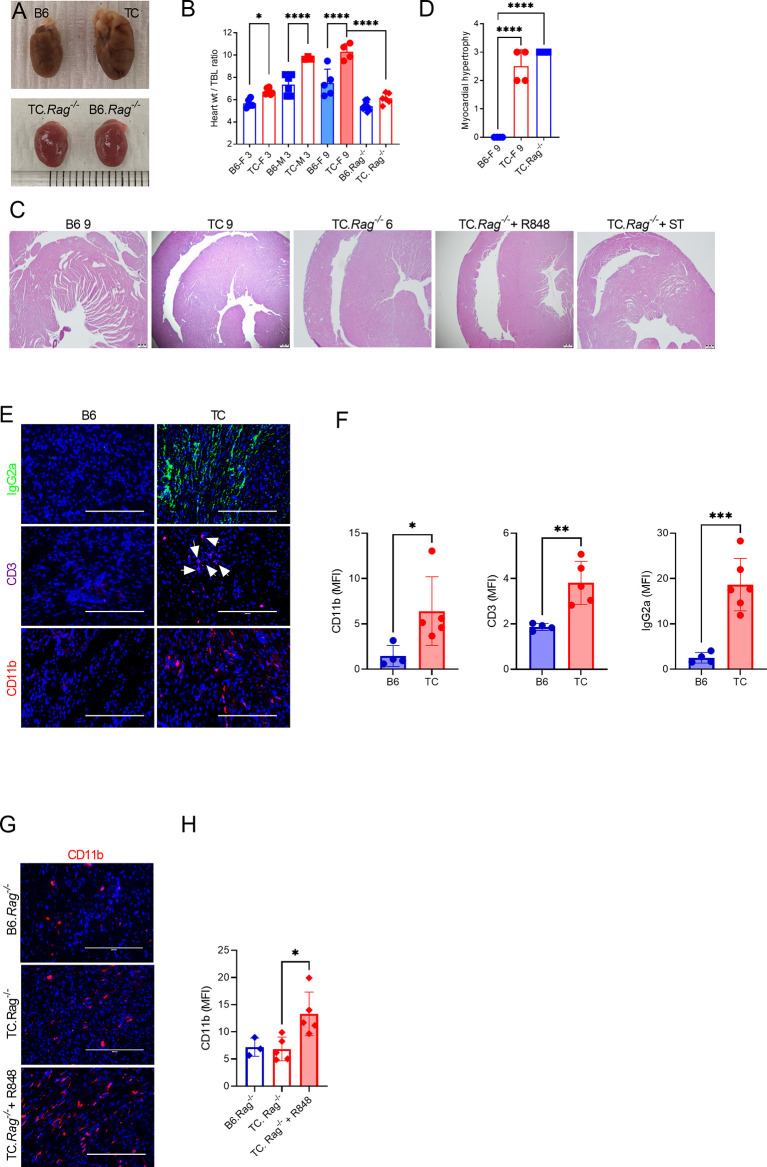
Both lymphoid and myeloid cells contribute to TC cardiovascular phenotypes. **(A)** Representative hearts from 9-months old B6 and TC mice and 6-months old B6.*Rag^-/-^* and TC.*Rag^-/-^* mice photographed at the same amplification. **(B)** Heart to tibia length ratios. **(C)** Representative H&E-stained heart sections (4X, scale bars: 200 um) of the indicative strain/conditions. **(D)** Myocardial hypertrophy scores. **(E)** Representative heart sections from 9 months old B6 and TC mice stained for IgG2a, CD11b and CD3 (20X, scale bars: 200 um). **(F)** Quantification of CD11b^+^ and CD3^+^ cells and IgG2a deposits (MFI). **(G)** Representative heart sections of 6-months old B6.*Rag^-/-^* and TC.*Rag^-/-^* mice, treated or not with R848, stained for CD11b (20X, scale bars: 200 um). **(H)** Quantification of CD11b^+^ cells (MFI). n = 3 - 10 mice per group, 1-way ANOVA, with multiple comparison tests, *: P < 0.05, **: P < 0.01, ***: P < 0.001. ****: P < 0.0001.

Serum and splenocytes adoptive transfers suggested that autoantibodies and activated immune cells trigger myocytolysis. Therefore, we assessed the presence of heart lesions in 9 months old TC females that produced high level of autoantibodies as compared to age-matched B6 females. Old TC mice have enlarged hearts compared to B6 (**Figures 7A, B**). Although untreated young TC mice did not show tissue injury or infiltrates, they also have enlarged hearts as measured by the heart weight to tibia length ratio compared with age and sex-matched B6 mice (**Figure 7B**). Only TC mice presented with moderate to severe myocardial hypertrophy (**Figures 7C, D**). These lesions were similar to those found in serum and splenocyte transfer recipients (**Figure 3B**). The heart of old TC mice also presented weak myeloid and T cell infiltrates as well as abundant IgG2a deposits (**Figures 7E, F**). These results suggest that cardiac hypertrophy occurs spontaneously in TC mice as a consequence of systemic autoimmunity.

The hearts of pre-autoimmune TC mice treated with R848 showed capillary injury, vascular congestion, and vascular dilatation (**Figure 1D**). However, these phenotypes were absent after serum and splenocyte transfer (**Figure 3A**) as well as in untreated autoimmune TC mice (**Figure 7C**). To test whether this vascular pathology required an acute TLR7 activation, TC with elevated levels of autoantibodies and age-matched B6 controls were treated with R848 for two weeks and evaluated one week later. Autoimmune treated TC mice showed severe capillary injury, vascular congestion, dilatation and myocytolysis as compared to treated B6 control (**Supplementary Figures S5A, B**). As with pre-autoimmune mice, CD45^+^ cell infiltrates were more abundant in the heart of autoimmune TC treated mice (**Supplementary Figure S5C**). These results indicate that acute TLR7 activation is required for cardiovascular injury

### Both Lymphoid and Myeloid Cells Contribute to Specific TC Cardiovascular Phenotypes

Since myeloid cells dominated the infiltrates in R848-treated TC hearts, we investigated whether the adaptive immune system was required for cardiac lesions in TC.*Rag^-/-^* mice, which lack T and B cells. TC.*Rag^-/-^* mice suffer a premature mortality as compared to TC mice for reasons that remain unclear (unpublished). Therefore, untreated TC.*Rag^-/-^* mice were evaluated at 6 months of age. The size of their heart was similar to that of intact 3 months old mice, with no difference between TC.*Rag^-/-^* and B6.*Rag^-/-^* mice (**Figures 7A, B**). This suggests that lymphoid cells contribute to TC spontaneously enlarged hearts. However, histological myocardial hypertrophy (**Figures 7C, D**) as well as a moderate CD11b^+^ myeloid infiltrate (**Figures 7G, H**) were observed in TC.Rag^-/-^ mice. No endothelial injury was identified in R848-treated TC.Rag^-/-^ mice (**Figure 7C**), in contrast to treated TC mice (**Figure 1D**). Furthermore, as with TC recipients, TC.*Rag^-/-^* recipients of serum from treated TC mice did not present any endothelial injury (**Figure 7C**). R848 treatment, however, enhanced CD11b^+^ myeloid cell infiltration in the heart of TC.*Rag^-/-^* mice (**Figures 7G, D**). Interestingly, no further aggravation of myocardial hypertrophy was detected as compared with untreated TC.*Rag^-/-^* mice (**Figure 7C**). These results suggest that T or B cells are not required for TC mice to develop reactive myocardial hypertrophy, but they are required for the TLR7-induced vascular injury.

**Figure 8:**
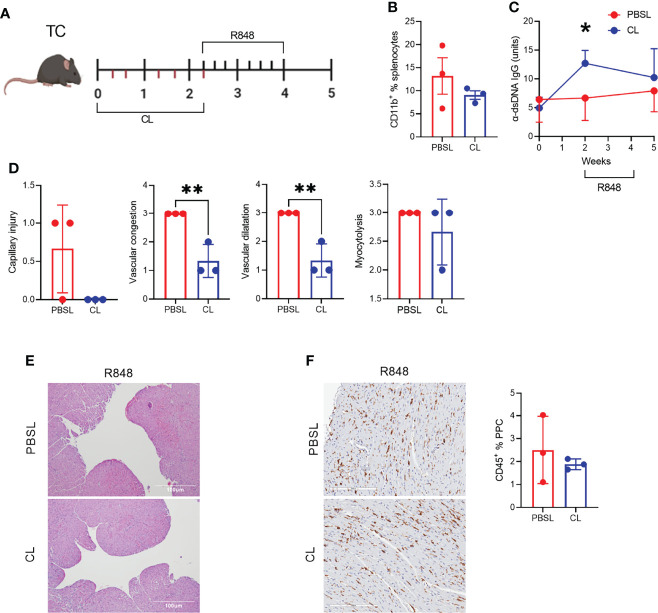
CD11b^+^ cell depletion decreased TLR-7-induced vascular injury. **(A)** Experimental design for pre-treatment with clodronate liposomes (CL) or PBS-loaded control liposomes (PBSL) before R848 treatment of pre-autoimmune TC mice. The time scale is in weeks and each tick corresponds to an injection of liposomes (bottom) or a R848 application (top). **(B)**. Frequency of CD11b^+^ splenocytes. **(C)** Serum anti-dsDNA IgG starting at the time of CL/PBSL treatment. **(D)** Cardiac pathology scores. **(E)** Representative H&E-stained heart sections of TC mice treated with PBSL or CL (10X, scale bars: 100 um). **(F)** CD45 staining with representative sections on the left (20X magnification, scale bar: 200 um) and quantitation of the right. n = 3 per group, t tests, *: P < 0.05, **: P < 0.01.

To further investigate the role of CD11b^+^ myeloid cell infiltrates in the heart after R848 treatment (**Figures 2A, B**), we used clodronate (CL) liposomes, which have been used to deplete phagocytic cells in lupus-prone mice (45). Pre-autoimmune TC mice were treated with CL or PBS-loaded control (PBSL) liposomes over two weeks before the R848 treatment with one injection at the time of the first R848 application (**Figure 8A**). The frequencies of total CD11b^+^ cells (**Figure 8B**) as well as neutrophils monocytes and macrophages numbers (data not shown) tended to be lower in CL-treated TC mice, but they had started to recover nearly three weeks after the last CL treatment. TC mice showed a transient production of anti-dsDNA IgG after two weeks of CL injection compared to PBSL controls (**Figure 8C**), corresponding to the reported disease acceleration in CL-treated lupus-prone mice (46). The hearts of CL-treated TC mice showed decreased capillary injury, congestion and dilatation (**Figures 8D, E**). However, myocytolysis and the CD45^+^ hematopoietic cell infiltrate were less affected by the depletion (**Figures 8D, F**). Overall, these results suggest that TLR7-induced capillary injury requires the presence of CD11b^+^ cells.

## Discussion

Here we report that acute TLR7 activation induces three types of lesions in the heart of lupus-prone TC mice: acute microvascular inflammation, myocytolysis with intracellular vacuolization and reactive myocardial hypertrophy. To our knowledge, this is the first report of cardiac microvascular inflammation in a lupus model. The endothelial cell injury and the presence of intra-capillary activated leucocytes were the most salient manifestations in the heart of R848- treated TC mice, which shared many histopathology features with AMR in allograft transplantation (47). AMR is triggered by anti-HLA alloantibodies binding to the endothelium on the transplanted heart, causing myocardial injury through both complement-dependent and independent pathways (37, 48). Complement-dependent immune complexes are well-known mediators of tissue inflammation including lupus nephritis. In complement-independent mechanisms, NK cells, macrophages and neutrophils may bind to antibody-bound endothelial cells *via* Fc receptors and enhance the inflammatory milieu through cytokine release and cytotoxicity (48). R848-treated TC mice produce autoantibodies with heart tissue reactivity as well as anti-phospholipid antibodies, which bind directly the endothelium (49). Accordingly, an abundant IgG2a deposition as well as a homogenous myeloid infiltrate were found in the heart of treated TC mice, but complement C3 and C4d were not detected. These results suggest that autoantibody deposition may initiate a complement-independent AMR, in which myeloid cells interact with antibody-bound endothelial cells *via* Fc receptors.

Edema, vessel wall damage and red blood cell extravasation found in the heart of R848-treat TC mice are shared features with the R848-induced myocarditis in non-autoimmune CFN mice (23). However, the inflammation in treated TC mice was largely confined to the microvasculature, while treated CFN mice presented cardiomyocyte necrosis, scattered interstitial immune cell infiltration into tissues and subsequent fibrosis. In other words, the inflammation targeted mainly endothelial cells in TC mice, and mainly cardiomyocytes in CFN mice. Importantly, immune cell infiltrates were dominated by B and T cells in treated CFN hearts (23), while the infiltrates in treated TC hearts consisted largely of myeloid cells with some T cells, but no B cells. The absence of microvascular inflammation in treated TC.*Rag^-/-^* mice suggests that it requires lymphocytes. We confirmed that R848 induced only very mild lesions in B6 mice (23). Therefore, acute TLR7 activation causes different heart histopathology in different genetic backgrounds likely through different mechanisms.

Adoptive transfer of serum or activated splenocytes from treated TC mice induced myocytolysis and reactive myocardial hypertrophy, but not capillary injury. This result suggests that microvascular injury requires a direct TRL7 activation on the endothelium. Since microvascular inflammation was not detected in the heart of R848 treated TC.*Rag^-/-^* mice or in TC mice in which myeloid cells have been depleted, T cells and/or B cells (or the autoantibodies they produce) as well as myeloid cells are required for acute microvascular inflammation. Taken together, our results suggest that R848 treatment of TC mice initiates the production of autoantibodies, some of which target the cardiac endothelium, which, combined with a direct TLR7 activation of the endothelium, as well as inflammatory signals produced by infiltrating myeloid cells, induced microvascular inflammation with an AMR-like pathology. The lack of difference between serum and splenocytes transfer also supports this model, with autoantibodies, chemokines and cytokines either secreted by transferred activated splenocytes or already in the transferred serum. R848 activates intravascular monocytes and triggers the endothelium to produce fractalkine and other pro-inflammatory mediators, which promote the intravascular retention of monocytes (41). These monocytes in turn recruit neutrophils, which mediate a focal necrosis of endothelial cells. Finally, the resulting cellular debris are phagocytosed by monocytes. Increased levels of necrosis and defects of the clearance of cellular debris contribute to the initiation of autoimmunity in lupus (50), and may play a role in the microvascular inflammation.

Based on a model developed for TLR-induced lupus nephritis (39), we propose that acute TLR7 activation activates intravascular PMos and triggers the endothelium to express increasing amounts of adhesion molecules and chemokines, which promote prolonged contact with PMos. Intravascular retention of PMos recruits neutrophils, which cause focal endothelial cells necrosis (41). In the B6 non-autoimmune background, monocytes scavenge cellular debris and prevent further inflammation. In the lupus-prone background and its over-activated immune response, increased levels of necrosis and defects in cellular debris clearance fuel the production of autoantibodies targeting endothelial autoantigens, and the development of microvascular inflammation in an AMR-like pathology. We have detected CD43^+^CD11b^+^ cells in the heart of treated TC mice at several time points during and after the R848 treatment. The exact role that these PMos play in the initiation and maintenance of the cardiovascular lesions will require additional studies in parallel with their better-defined function in lupus nephritis. We hypothesize that the interstitial capillary injury is central to the cardiovascular response of lupus-prone mice to acute TLR7 activation as prolonged R848 treatments results in fatal hemorrhages in TC mice. This mechanism of microvascular injury may contribute, at least in part, to myocardial dysfunction in lupus patients through poor perfusion.

Myocytolysis with intracellular vacuolization was a- feature of both R848-treated non-autoimmune (CFN and B6) and autoimmune (TC) mice. This process usually starts around apparently normal nuclei with myofibrillar disappearance producing an increasing vacuolization the myocardial cells resulting ultimately in empty sarcolemmal tubes (51). This reversible ischemic lesion is seen in congestive heart failure as well as in any heart not adequately perfused (52). Perfusion abnormalities have been detected by SPECT imaging in 88% of SLE patients, two thirds of which had negative coronary angiograms (53), which is in agreement with the reduction of myocardial coronary flow reserve on MRI studies found in 44% of SLE patients with angina and a normal angiogram (53). These findings suggest that coronary microvascular dysfunction, which has emerged as a mechanism of myocardial ischemia, heart failure and arrhythmias distinct from obstructive atherosclerosis, is a common feature in SLE patients. Further, myocytolysis due to inadequate heart perfusion may connect microvascular and myocardial dysfunctions.

Since an acute microvascular inflammation was observed only in R848-treated TC mice, microvascular inflammation is not required for myocytolysis. Mild to moderate myocytolysis was induced in TC recipients of serum or splenocytes from treated TC mice. In addition, myocytolysis and reactive myocardial hypertrophy developed in older TC and TC.*Rag^-/-^* mice, both of which share an activated innate immune system, but only the former produce autoantibodies. This suggests that other factors, such as inflammatory cytokines may induce poor perfusion. Myocyte loss is coupled with a progressive increased volume of the remaining still viable cells, a process named reactive cellular hypertrophy, a common pathological feature after a myocardial infarction (54) and age-related myocardiocyte loss (55, 56). Interestingly, spontaneous mild myocytolysis was visible in old B6 and TC mice, but only old TC mice presented with moderate to severe myocardial hypertrophy. Myocardial hypertrophy was associated with myocytolysis in all autoimmune-activated TC mice in this study, which suggested that blood-borne factors and/or immune cell activation may be required for myocardial hypertrophy. Myocytolysis and reactive cellular hypertrophy are detected only by histology, which is largely performed post-mortem. In a recent report, 9 out of 11 endomyocardial biopsies from SLE patients presented with myocyte hypertrophy (57), suggesting that the clinical importance of myocyte hypertrophy may be highly underestimated in SLE. Potential associations between myocardiocyte hypertrophy and heart failure or unexplained mortality in SLE patients remain to be explored. Taken together, we propose a model of reactive myocardial hypertrophy that occurs spontaneously in TC mice as a consequence of systemic autoimmunity. Lymphocytes contribute to TC spontaneously enlarged hearts as TC.Rag^-/-^ mice have smaller hearts. Either activated immune cells or blood-born factors could induce reactive myocardial hypertrophy, however, neither T or B cells nor autoantibodies were required for TC mice to develop reactive myocardial hypertrophy. TLR7 activation accelerated full-blown lupus in young lupus prone TC mice characterized by the acute activation of innate and lymphoid cells, which accelerated cardiac hypertrophy.

In summary, we report a novel model of lupus-associated cardiovascular pathology induced by R848 treatment that is characterized by an acute autoimmune microvascular inflammation and myocytolysis with intracellular vacuolization. Further, we report that reactive cardiomyocyte hypertrophy presents spontaneously in autoimmune TC and TC*.Rag^-/-^* mice or in recipients of serum or splenocytes transferred from R848 treated lupus-prone TC mice. Each of these cardiovascular lesions correspond to abnormalities that have been reported in SLE patients, which can now be investigated using the TC model with R848 as well as the adoptive transfer of immune cells or serum factors.

## Data Availability Statement

The original contributions presented in the study are included in the article/**Supplementary Material**. Further inquiries can be directed to the corresponding author.

## Ethics Statement

The animal study was reviewed and approved by the Institutional Animal Care and Use Committee of the University of Florida (IACUC 202009466).

## Author Contributions

AE, XT and LM designed the experiments and analyzed results. AE, NK, WL, S-CC and GA conducted experiments. AE and MT performed tissue processing and immunofluorescence staining and analysis. Histology was reviewed by XT and KF and scored by KF. AE, XT, KF and LM wrote the manuscript. All authors contributed to the article and approved the submitted version.

## Conflict of Interest

The authors declare that the research was conducted in the absence of any commercial or financial relationships that could be construed as a potential conflict of interest.

## Publisher’s Note

All claims expressed in this article are solely those of the authors and do not necessarily represent those of their affiliated organizations, or those of the publisher, the editors and the reviewers. Any product that may be evaluated in this article, or claim that may be made by its manufacturer, is not guaranteed or endorsed by the publisher.
