# Supplementary material for: TLR7 Activation Accelerates Cardiovascular Pathology in a Mouse Model of Lupus
Source: Front Immunol. 2022 Jul 4;13:914468. doi: 10.3389/fimmu.2022.914468 (PMC9289616; doi:10.3389/fimmu.2022.914468)
Supplement: Supplementary file 1 [file DataSheet_1.pdf]

Table S1. Primer sequences

	Forward	Reverse
Ppia	CACAGCCAAGGGTCGATTCC	CCCAGGTATCGTGCTTTGTCT
Isg15	GAGCTAGAGCCTGCAGCAAT	TAAGACCGTCCTGGAGCACT
Irf7	CAGCGAGTGCTGTTTGGAGAC	AAGTTCGTACACCTTATGCGG
Ifng	GAGCTCATTGAATGCTTGGC	GCGTCATTGAATCACACCTG
Tnfa	CCACCACGCTCTTCTGTCTAC	AGGGTCTGGGCCATAGAACT
