# Supplementary material for: TLR7 Activation Accelerates Cardiovascular Pathology in a Mouse Model of Lupus
Source: Front Immunol. 2022 Jul 4;13:914468. doi: 10.3389/fimmu.2022.914468 (PMC9289616; doi:10.3389/fimmu.2022.914468)
Supplement: Supplementary file 2 [file DataSheet_2.pdf]

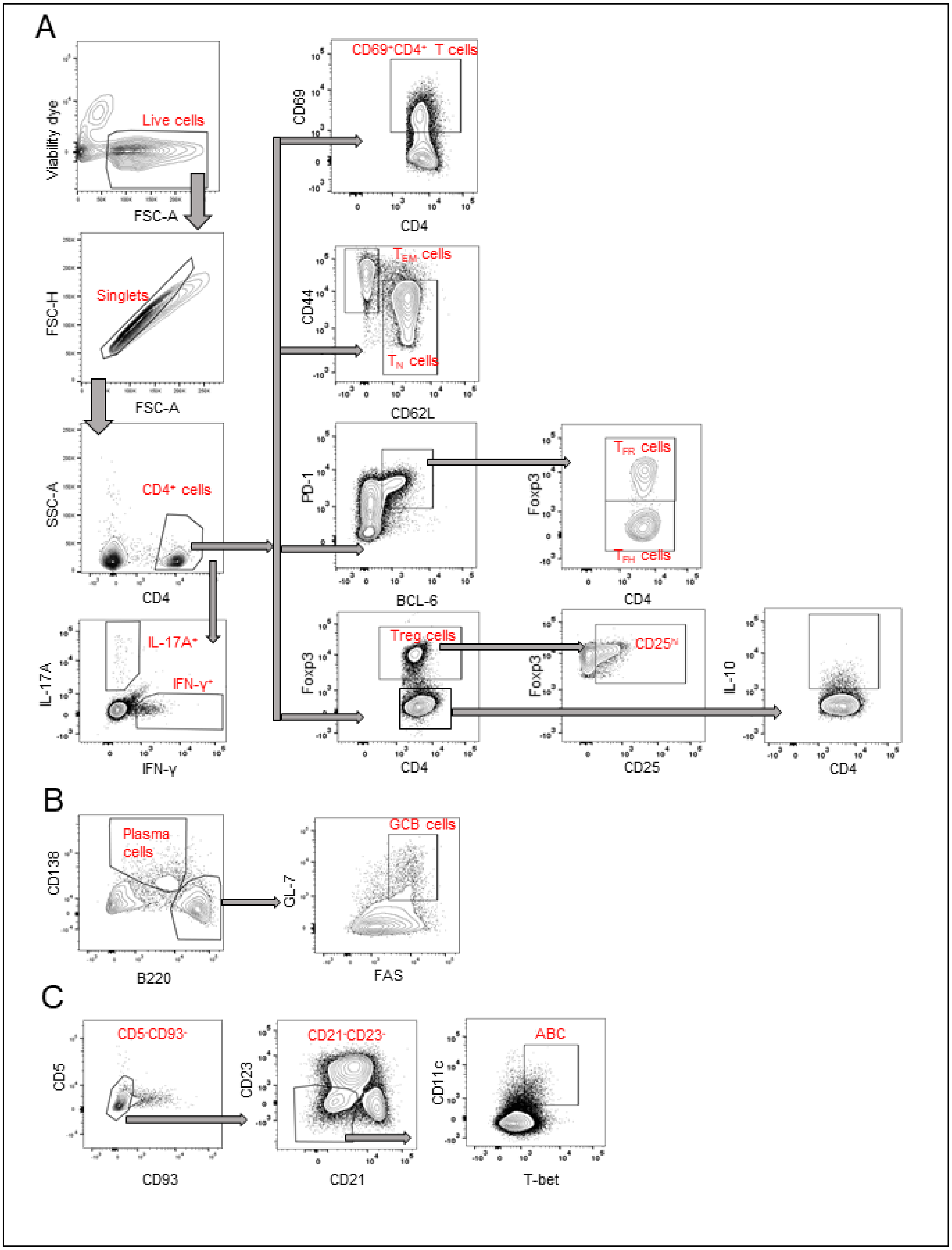

Figure S1. T and B cell gating schemes. A. $CD4^+$ T cells. **B**. Plasma cells and germinal center B cells. **C**. Class-switched B cells and ABC B cells.

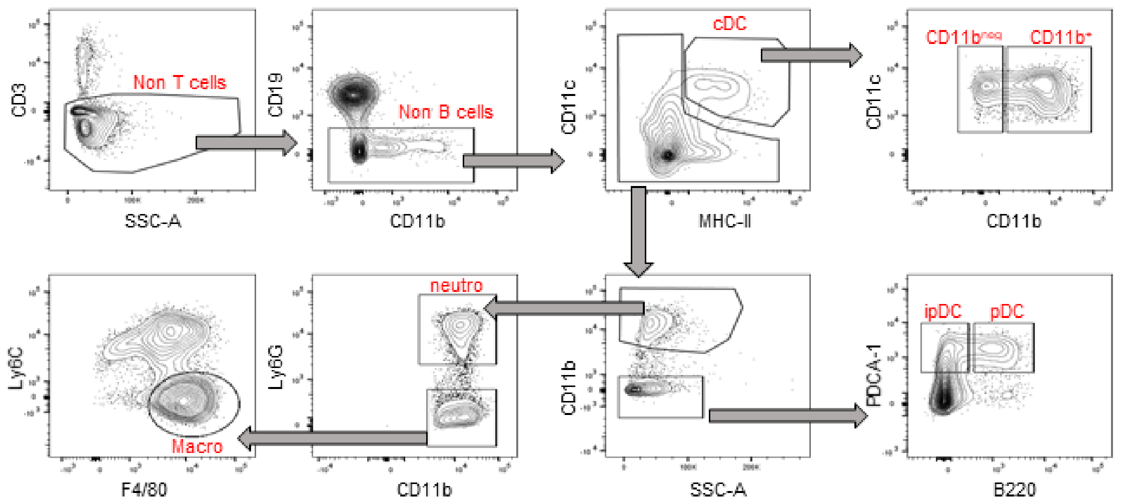

Figure S2. Myeloid cell gating schemes

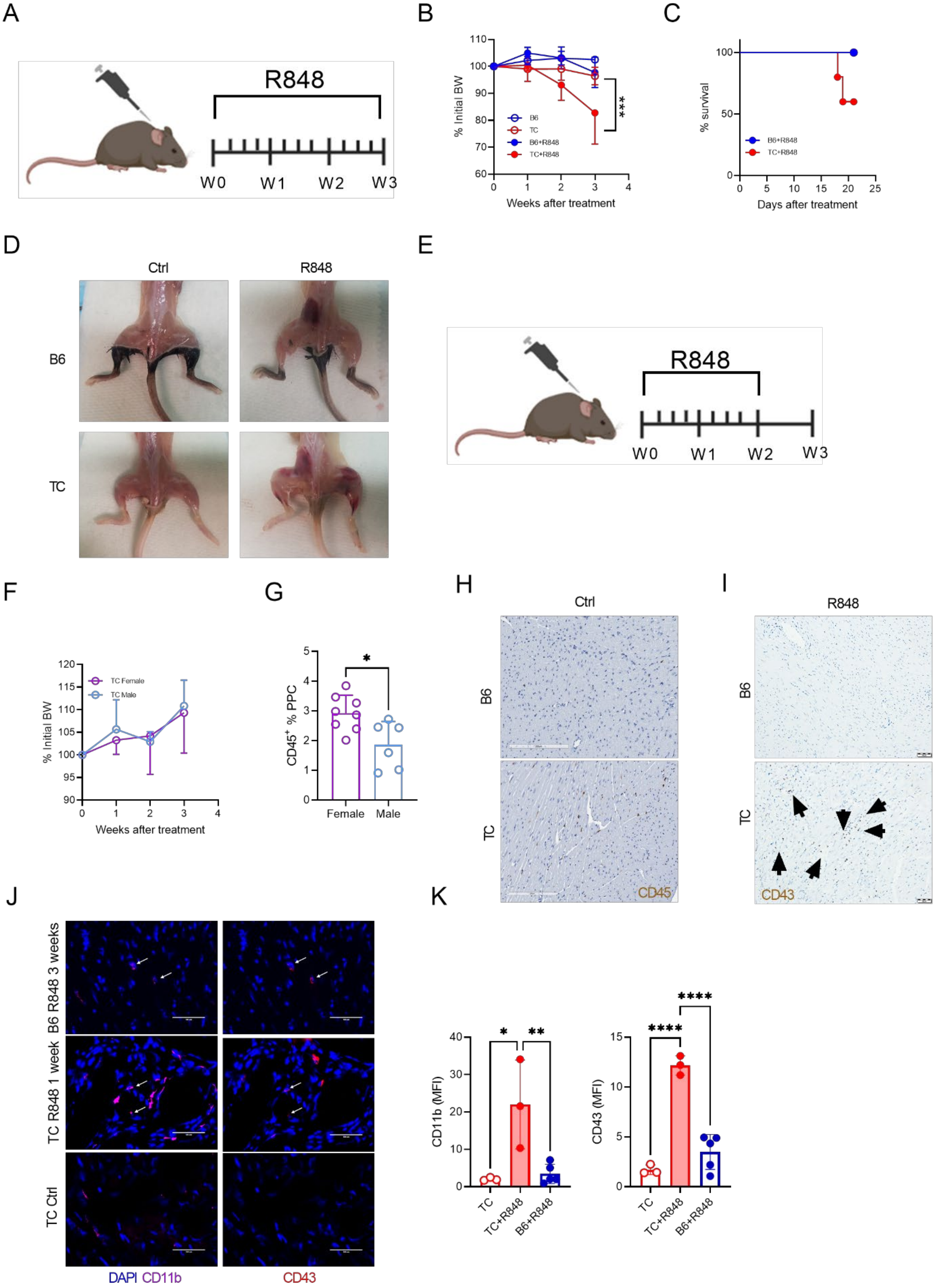
A
R848
W0
W1
W2
W3
B
% Initial BW
Weeks after treatment
B6
TC
B6+R848
TC+R848
***
C
% survival
Days after treatment
B6+R848
TC+R848
D
Ctrl
R848
B6
TC
E
R848
W0
W1
W2
W3
F
% Initial BW
TC Female
TC Male
Weeks after treatment
G
CD45+ % PPC
*
Female
Male
H
Ctrl
B6
TC
CD45
I
R848
B6
TC
CD43
J
B6 R848 3 weeks
TC R848 1 week
TC Ctrl
DAPI CD11b
CD43
K
CD11b (MFI)
*
**
TC
TC+R848
B6+R848
CD43 (MFI)
****
****
TC
TC+R848
B6+R848

Figure S3. Phenotypes induced by R848 in TC and B6 mice. (**A**) Experimental design of the 3-week R848 treatment in two month old mice. The time scale is in weeks and each tick corresponds to a R848 application (top). (**B**) Weekly body weight shown as percent of initial weight in mice treated or not with R848 (2-way ANOVA). (**C**) Survival of treated mice. (**D**) Representative images showing extensive hemorrhages in TC females after 3 weeks of treatment. (**E**) Experimental design of the 2-week R848 treatment of two month old mice with sacrifice at the end of week 3). (**F – G**) Body weight (**F**) and quantification of CD45 staining in the heart (**G**). TC females and males. (**H**) Representative CD45 staining (brown) in untreated B6 and TC mice (20X magnification, scale bar: 200 um). (**I**) Representative heart sections stained with CD43 (brown) after 3 weeks of treatment (20X, scale bars: 50 um). (**J**) Representative CD11b (left) and CD43 (right) in the heart of, from top to bottom, a B6 mouse after 3 weeks of treatment, a TC mouse after 1 week of treatment, and an untreated TC mouse (40x, scale bars: 100 um). (K) Quantification of CD11b+ and CD43+ infiltrates (MFI). Arrows indicate co-staining. *t* tests, * $P < 0.05$, *** $P < 0.001$. **A - D, F - H**: n = 5 females per group, **F - G**: n = 8 females and 6 males.

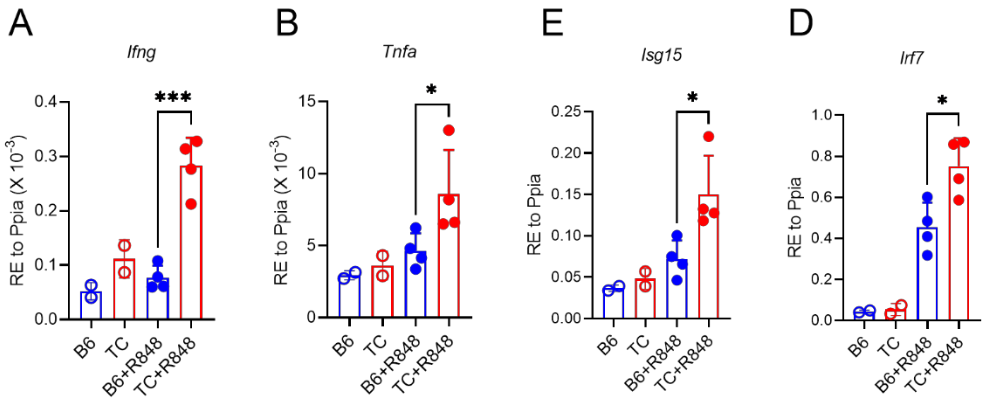

Figure S4. Gene expression in the heart of pre-autoimmune B6 and TC mice treated or not with R848. Relative expression of *Ifng* (**A**), *Tnfa* (**B**), *Isg15* (**C**) and *Irf7* (**D**). n = 2 - 4 per group. *t* tests, *: P < 0.05, ***: P < 0.001.

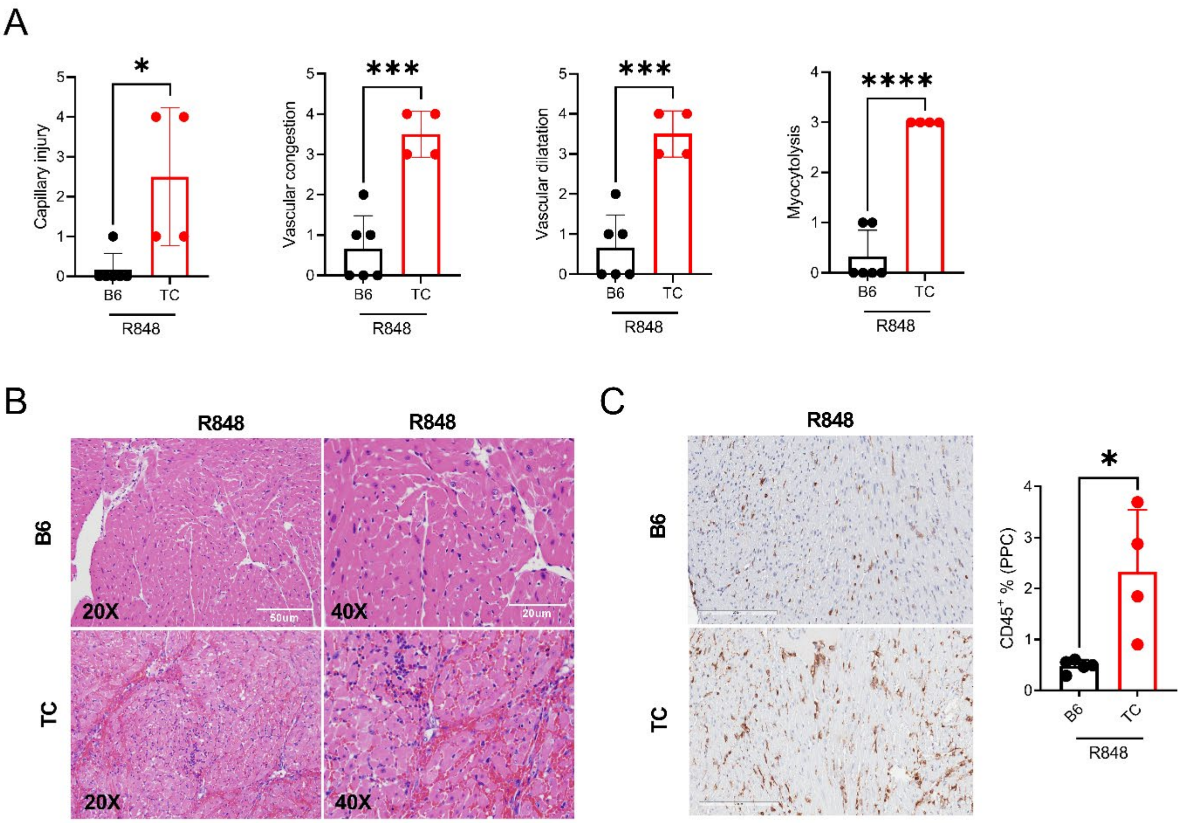

Figure S5. TLR7 activation in autoimmune TC mice and B6 controls. Anti-dsDNA IgG positive 10 months old TC mice and B6 controls were treated with R848 for two weeks and evaluated one week later. (**A**) Capillary injury, vascular congestion, vascular dilatation and myocytolysis scores compared by *t* tests. (**B**) Representative H&E-stained heart sections (20X and 40X magnification (scale bars: 50 um and 20 um, respectively). (**C**) CD45 staining with representative sections on the left (20X magnification, scale bar: 200 um) and quantitation of the right. N = 4 - 6 per group, * $P < 0.05$, *** $P < 0.001$, **** $P < 0.0001$.

.
